# In it for the long haul: the complexities of managing overweight in family practice: qualitative thematic analysis from the Health eLiteracy for Prevention in General Practice (HeLP-GP) trial

**DOI:** 10.1186/s12875-023-01995-w

**Published:** 2023-02-27

**Authors:** Katrina Paine, Sharon Parker, Elizabeth Denney-Wilson, Jane Lloyd, Sue Randall, Carmel McNamara, Don Nutbeam, Richard Osborne, Shoko Saito, Mark Harris

**Affiliations:** 1 Susan Wakil School of Nursing, Faculty of Medicine and Health, University of Sydney, Camperdown, Australia; 2 Centre for Primary Health Care and Equity, Faculty of Medicine and Health, University of New South Wales, Kensington, Australia; 3 Susan Wakil School of Nursing, Faculty of Medicine and Health, University of Sydney and Sydney Local Health District, Camperdown, Australia; 4 Susan Wakil School of Nursing, Faculty of Medicine and Health, University of Sydney, Camperdown, Australia; 5 College of Nursing and Health Science, Flinders University, Adelaide, Australia; 6 Public Health in the School of Public Health, Faculty of Medicine and Health, University of Sydney, Camperdown, Australia; 7 Centre of Global Health and Equity, Swinburne University of Technology, Melbourne, Australia; 8 Health Equity and Research Development Unit, Faculty of Medicine and Health, University of New South Wales, Kensington, Australia

**Keywords:** Family practice, Managing overweight, Obesity, Patient-provider relationships, Primary care, *mySnapp*, Get healthy telephone coaching

## Abstract

**Background:**

Australia has one of the highest rates of overweight and obesity in the developed world, and this increasing prevalence and associated chronic disease morbidity reinforces the importance of understanding the attitudes, views, and experiences of patients and health providers towards weight management interventions and programs. The purpose of this study was to investigate patients, family practitioners and family practice nurses’ perceptions and views regarding the receipt or delivery of weight management within the context of the HeLP-GP intervention.

**Methods:**

A nested qualitative study design including semi-structured interviews with family practitioners (*n* = 8), family practice nurses (*n* = 4), and patients (*n* = 25) attending family practices in New South Wales (*n* = 2) and South Australia (*n* = 2). The patient interviews sought specific feedback about each aspect of the intervention and the provider interviews sought to elicit their understanding and opinions of the strategies underpinning the intervention as well as general perceptions about providing weight management to their patients. Interviews were recorded and transcribed verbatim, and coding and management conducted using NVivo 12 Pro. We analysed the interview data using thematic analysis.

**Results:**

Our study identified three key themes: long-term trusting and supportive relationships (being ‘in it for the long haul’); initiating conversations and understanding motivations; and ensuring access to multi-modal weight management options that acknowledge differing levels of health literacy. The three themes infer that weight management in family practice with patients who are overweight or obese is challenged by the complexity of the task and the perceived motivation of patients. It needs to be facilitated by positive open communication and programs tailored to patient needs, preferences, and health literacy to be successful.

**Conclusions:**

Providing positive weight management in family practice requires ongoing commitment and an open and trusting therapeutic relationship between providers and patients. Behaviour change can be achieved through timely and considered interactions that target individual preferences, are tailored to health literacy, and are consistent and positive in their messaging. Ongoing support of family practices is required through funding and policy changes and additional avenues for referral and adjunctive interventions are required to provide comprehensive weight management within this setting.

**Supplementary Information:**

The online version contains supplementary material available at 10.1186/s12875-023-01995-w.

## Background

Most of the world’s population live in countries where overweight and obesity kills more people than underweight [1]. Australia has one of the highest rates of overweight and obesity in the developed world [2] with rates doubling over the past two decades, and 67% of adults affected [2, 3]. The increasing prevalence of overweight and obesity in Australia and associated chronic disease morbidity reinforces the importance of understanding the attitudes, views, and experiences of patients and health providers towards weight management interventions and programs.

Within Australia, general practice (GP) or family practice is an important contributor to treatment and prevention of overweight and obesity. The term family practice is used in this publication as this term has wider recognition from an international perspective. Participants attending primary care view their family medical practitioner (FP) and family practice nurse (FPN) as having a key role in managing obesity [4, 5]. We know from previous research however that within this setting weight, body mass index (BMI) and waist circumference (WC) are infrequently assessed [6–8], and opportunities to provide comprehensive weight management advice is often missed [6–8]. Moreover, there are challenges in delivering preventive care for weight management reflecting its complex, variable, and time-consuming nature.

Obesity is a chronic and relapsing disease [9]. Medically the concept of weight homeostasis with upregulation of physiological pathways leading to hunger and a diminution of energy means that for many people, returning to a starting weight is likely [10]. We also live in a obesogenic environment characterised by continuous access to high-energy foods combined with reduced obligations for physical activity [11]. Obesity and the associated comorbidities come with significant psychosocial burden including stigma, depression and anxiety, eating disorders, substance abuse, poor body image and poor self-esteem [12]. Addressing weight in family practice is also influenced by provider skill, willingness and interest [13], as well as patient motivation, health literacy, and personal and social circumstances [14]. In addition, the structure of the Medicare Benefits Schedule (MBS) – Australia’s national health insurance scheme – provides better remuneration for multiple standard consultations compared to a single longer consultation, and there is a lack of specific incentives to help overweight and obese patients achieve healthy weight [15, 16].

The Health eLiteracy for Prevention in General Practice (HeLP-GP) cluster randomised controlled trial was conducted 2017-2020. Twenty-two family practices in lower socioeconomic areas of Sydney, New South Wales (NSW) and Adelaide, South Australia (SA) consented to participate and 11 were allocated to the intervention and 11 to usual care. Patients of participating practices were flagged at presentation using Doctors Control Panel (DCP) and approached to consent. In total 215 participants were recruited to the study (120 to the intervention group and 95 to the control group). The main outcomes of this study have been reported in Parker et al. [17]. This nested study used qualitative interviews to elicit patient and provider perceptions and views regarding the receipt or delivery of weight management within the context of the HeLP-GP intervention. It aimed to deepen our understanding of the study implementation and outcomes, as well as to provide practical insights to guide future interventions of this type.

## Methods

### Context

The HeLP-GP intervention supported overweight and obese participants by providing a FPN-led tailored health check based on the 5As model of patient-centred care: Ask, Advise, Assess, Assist, and Arrange [18], combined with a purpose-built lifestyle app (*mysnapp*) and/or referral to a free telephone coaching service (Get Healthy^1^) [19]. This combination intervention aimed to assist participants to improve their diet, increase their level of physical activity, and improve their general health.

Eligible participants were 40-74 years of age, overweight or obese (BMI ≥ 28), had their weight and blood pressure (BP) recorded within the previous 12 months, and had access to a smart phone or tablet device [19]. Participants had to be able to speak and read either English, Arabic, Chinese, or Vietnamese.

The HeLP-GP study (including the qualitative study) was approved by the University of New South Wales Human Research Ethics Committee (HC17474) and ratified by the University of Adelaide Human Research Ethics committee. All experiments were performed in accordance with relevant guidelines and regulations. This trial is registered with the Australian New Zealand Clinical Trials Registry (ACTRN12617001508369, date registered 26/10/2017). http://www.ANZCTR.org.au/ACTRN12617001508369.aspx

### Qualitative sample selection and recruitment

Invitations to participate were extended to four of eleven intervention practices (two from each participating state). FPs and FPNs were eligible for interview if they had been actively involved in delivering the HeLP-GP intervention and patients were approached only if they had attended the health check. Patients were invited to participate at the routine 6-month follow-up. The size of the practice (< 5 or ≥ 5 FPs) and the gender and age of patients were used to maximise our chances of recruiting a more diverse sample.

### Development of the interview guides

Three semi-structured interview guides were developed for each participant cohort (i.e., patients, FPs and FPNs). These guides were designed by a working group comprising trial investigators and research staff and were based on the findings of a preliminary study [20]. The interview guides were tailored for the participant group. Specifically, the wording of questions were refined and simplified based on feedback from the piloting phase. Some of the questions were broken into simpler questions to elicit a clearer response from the participant. The participant interviews sought specific feedback about each aspect of the intervention (i.e. *mysnapp* or Get Healthy telephone coaching) and provider interviews included questions to gauge their understanding and opinions of the underpinning strategies embedded in the intervention (the 5As and teach-back models). To frame the interview context, they all began with questions about preventive care to elicit participants’ understandings of the concept and general viewpoints about receiving or delivering preventive care. After piloting the interview guides on eight people, refinements were made based in this feedback.

The final conversation guides (see Appendixes A, B and C) followed a traditional structure characterised by a small number of open-ended questions with a series of specifically designed prompts. The rationale for conducting semi-structured interviews was that we considered the format to encourage interviewees to relax and open up, affording them a sense of primary control (as opposed to researcher control) over the issues and concerns given focus. Reporting in this study was guided by the Consolidated Criteria for Reporting Qualitative Research (COREQ) checklist [21] as seen in Appendix D.

### Data collection

Interviews occurred between 3 and 6 months post-intervention to ensure interviewees had time to engage fully in the interventions yet were sufficiently recent to assist recall. Consent for interview (including audio-recording) was obtained verbally at the time of interview. All interviews were conducted between July 2019 and April 2020. Patient interviews were conducted over the telephone. An interpreter was offered if the patient spoke primarily a language other than English. The average duration of patient interviews was 23 minutes (range 11-59 minutes). Although this average duration is relatively short for telephone-based interviews, it proved sufficient for data collection from patients given the highly focused aim of the research. No other methods to interview the patient participants was offered. All interviews were undertaken by two research staff to ensure consistency in the interview methods. Face-to-face interviews with FPs and FPNs were conducted at the practice by Authors CM or KP. The average duration of these interviews was 23 minutes (range 9-38 minutes).

### Data management and analysis

Interviews were recorded and transcribed verbatim, then imported to NVivo 12 Pro. We analysed the interview data using the thematic analysis method proposed by Braun and Clarke [22]. Initially, transcripts were coded by two independent researchers (Authors KP and SS) and reviewed with the aim to identify and discuss any coding discrepancies. A coding framework was created to define/describe each code. Throughout the analysis the codes were cross-checked and any interpretive differences (e.g. omissions or commissions of concepts/ themes) were resolved by agreement following a review by Authors KP and SP. An expert research working group (which met fortnightly) discussed the codes and emergent themes (Appendix E). The research working group consisted of Author ED-W, a health expert in mixed methods research in public health; Author SR, a health expert in qualitative research in primary health; Author DN, an expert in mixed methods research in social science and public health, and Author MH an academic general practitioner with expertise in primary health care and primary health care research.

## Results

The final sample for the provider interviews included 8 FPs and 4 FPNs (Table 1).

**Table 1:** Practice and provider characteristics

ID No.	Practice Details						
	Size of practice	No. of FPs interviewed	Age range (years)	FP gender (M/F)	No. of FPNs interviewed	FPN gender (M/F)	Age range (years)
17	< 5 FPs	2	1 = 65+ 1 = 55-64	M, M	1	M	Unknown
18	≥5 FPs	1	45-55	F	1	F	20-34
22	≥5 FPs	3	1 = unknown 2 = 35-44	M, M, M	1	F	45-54
25	≥5 FPs	2	1 = 44-64 1 = 65+	M, M	1	F	35-44

Twenty-five patients were interviewed. Participants in the qualitative study had broadly similar demographics to those in the intervention arm of the main study Tables 2 and 3. Most were born in Australia (68%), and all chose to be interviewed in English. Within our sample roughly the same were male and female although in two practices we recruited no men for the qualitative interview. However, recruited numbers were lower in these two practices overall.

**Table 2:** Patient characteristics

Patients Interviewed for the Qualitative Study				
Practice ID.	No. of patients interviewed (%) at baseline	No. of females interviewed (%)	No. of males interviewed (%)	Mean age/age range of interviewees
17	14/35 (40)	6/19 (31)	8/16 (50)	56 (48-68)
18	6/22 (27)	1/11 (9)	5/10 (24)	56 (46-72)
22	1/7 (29)	½ (50)	0/5 (0)	58 (48-68)
25	4/11 (36)	4/6 (100)	0	68 (61-72)
Total	25 (34)	12 (46)	13 (52)	58 (48-72)
NSW Intervention	20 (20)	7 (7)	13 (13)	56 (46-72)
SA Intervention	6 (26)	5 (20)	0 (0)	63 (48-72)

**Table 3:** Baseline characteristics (intervention group): Full cohort and qualitative cohort

Variables	Responses	Full cohort (Intervention) n (%)	Qualitative cohort n (%)
No.		121	25
Age, mean (SD)		59.0 (8.8)	57.8 (8.9)
Gender	Female	60 (49.6)	13 (52.0)
	Male	61 (50.4)	12 (48.0)
Place of birth	Australia	66 (54.5)	17 (68.0)
	Overseas	55 (45.5)	8 (32.0)
Primary language at home	English	97 (80.2)	23 (92.0)
	Other	27 (19.8)	2 (8.0)
State	NSW	100 (82.6)	20 (80.0)
	SA	20 (17.4)	5 (20.0)

**Fig. 1:**
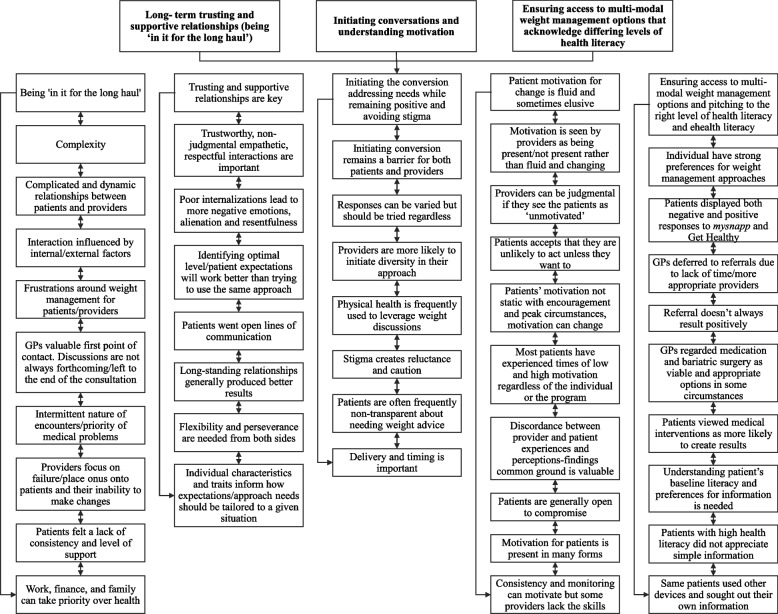
Three key themes and their inter-relationships with the five subthemes

We identified the following key themes from our data. Although we address these themes separately in the following section, substantial crossover and inter-relationships between themes were identified. Figure 1 shows the three key themes and identifies the inter-relationships with the subthemes.

### Long-term trusting and supportive relationships (being ‘in it for the long haul’)

This theme relates to the complexity of weight management and the gains made through interactions based on trust and empathy. Providers exhibited frustration and found engaging patients difficult, particularly with what they perceived to be entrenched and unmoving patterns of thinking and behaviour. The repetitiveness of trying to engage patients with weight management strategies was frequently perceived as requiring an investment they could not always offer, taking up time they did not often have, or as unfulfilling and a wasted effort:

*Weight is one of the most frustrating things you deal with. … Probably around two to 5 % (of patients) will lose weight over two years. They’ll lose it quickly. But then they put it back on again.* (FP, Male, 65+).

Likewise, patients expressed recurrent experiences when managing weight:

*… … .. I’m a Weight Watchers veteran, you know. I’ve done Weight Watchers on and off for years, and been on diets for years...* (Patient, Female, 54).

Despite seeing weight management and prevention as part of their clinical role, providers felt overwhelmed by the extent of the task within their practice and the expectations of patients. This created a reluctance to explore weight issues with some patients, particularly in the context of prioritising the patient’s presenting condition:

*… … I would say more than 50% of our patients have an overweight or obesity problem … ... But of course, when you bring it up, people say, oh okay doctor, how am I going to lose weight? So, suddenly it’s my fault, my problem, and I’ve got to make them lose weight, and so I can see why some doctors don’t bring it up. And then half an hour later you’re going round and round in circles with somebody because they say they can’t. But it has to be brought up. If you’ve already dealt with five different issues in five different parts of the body and then you’re suddenly telling them how to lose weight, well …* (FP, Male, 55-64).

There was a tendency for providers to express their interactions with patients using negative language rather than providing examples of ‘success’ and also to place the onus of weight management onto patients, or to focus blame on the patient’s lack of enthusiasm or inability to follow through or ‘stick to a program’:

*I find the ones that have the overweight issues, the story’s always the same: ‘I don’t want to move’, ‘It hurts’; and I realise I’m overweight but, um, it can’t hurt this bad, and things like that. All the excuses.* (FPN, Female, 35-44).

Similar to clinicians, patients’ attitudes and perspectives were moulded by past experiences and the feelings that manifest because of those experiences. Some expressed frustration with health professional they had perceived to treat them poorly, provide guidance lacking instruction, or not provide suitable tools to help them follow through:

*They do assessment and they say, do diet and exercise, and you relapse and don’t do it. They say to stay away from these foods, and if you don’t, you know, it’s useless even going to the FP.* (Patient, Male, 67).

Patients were more likely to be responsive to weight management advice when they perceived their provider to be trustworthy, non-judgemental, empathetic, and respectful. Within this study, this was true regardless of the provider (FPNs, FPs, health coaches etc.). When patients felt supported and comfortable in their interactions, they used words such as ‘openness’, ‘information sharing’, ‘honest’, ‘nurtured’, and ‘supported’. In addition, they were more inclined to engage in positive dialogue and weight management and lifestyle activities. This positive milieu was one in which the patient and provider could jointly plan or set goals and patients reflect on achievements/failures without fearing reprisal or judgement.

*Well, the guy (Get Healthy coach) had rung me weekly at first and then fortnightly. Now it’s about every third week, but honestly, I look forward to the calls because I can say to him, ‘Oh, I’ve lost this much’ or “I’ve lost weight around …’ you know, that sort of thing. But it just gave me someone to look forward to telling what I’ve done. It’s just really exciting when you’ve got the phone call and he’d be advising me on what I should do and what I could do and asking me questions about myself.* (Patient, Female, 63).

Patients who did not experience this safe and supportive relationship tended to express negative emotions. They described feeling alienated within the interaction (despite the content), misunderstood, and not listened to. These patients often discontinued using the program and exhibited reduced enthusiasm and engagement:

*My FP sent me to someone about going on a diet. I thought, ‘Yeah, I will go and see what it’s like, and yeah, I didn’t like it. He tried to tell me to pick and eat just one piece of fruit and that just turned me off. … So, he wasn’t listening to me* (Patient, Female, 57).

Patients preferred open lines of communication and mutual respect. These conditions emerged as the cornerstones of constructive dialogue and empowered patients to make their own health decisions and to be responsible for their actions:

*I feel they’re (FP and FPN) … incredibly courteous in the way they deal with me. I guess they speak to me very openly and I understand what they’re saying. I really appreciate that I’m part of it. They have stuff that they know, but they involve me in that information [so] I can have some ownership of it I don’t like being told what to do. They don’t tell me what to do, they give me information and we talk about it. I love that.* (Patient, Female, 67).

Many patients described consistent and long-standing relationships with their FPs (sometimes decades). This strengthened their belief that the health information and healthcare they received were both accurate and appropriate and these patients were more likely to join the study because the FP had suggested it:

*He [FP] generally looks at what I go there for, but he also weighs me, and we talk, just in general, not always about what I go there for. … He was the one that put me forward for this study.* (Patient, Male, 53).

Flexibility and perseverance were other characteristics promoting good interactions between providers and patients. In addition, the ability to engage in innovative interactions produced positive outcomes. Sometimes frequent and multiple approaches were needed to engage patients appropriately and to identify different interventions to best fit with their lifestyles. A FPN and FP, respectively, described their experiences with patients:

*I think he was feeling like it was such a rigid thing. I said, no, we don’t have to do it in that timeframe, we can do it whenever. … I really had to think outside the box to find a way to make it work for him and so, that really challenged me actually.* (FPN, Female, 45-54).

*Depending on how sensitive they are. If they are then I back off, if not I can try to tweak the conversation a bit and ask more in terms of the more general stuff, like, what they feel about their eating, or what they think they could improve on, rather than direct questions.* (FP, Male, Unknown).

### Initiating conversations and understanding motivations

Open discussions of weight between patients and clinicians resulted in a minefield of mixed opinions, sentiments, and pre-conceived beliefs. Patients and clinicians alike identified barriers to having conversations about weight:

*Some patients don’t really like us to point out that they are obese or they’re overweight. They’re not quite comfortable talking about their diet or their exercise so we just try to tell them but some of them just don’t take the advice.* (FPN, Female, 45-54).

For many patients, it was not the conversation that was most important but the way in which the message was conveyed:

*It just depends on how it’s delivered to you, or how someone brings it up as a subject. I think that’s going to be the problem with a lot of people; you know, ‘are you saying I’m fat?’...So, it’s got to be delivered in such a way that I think the person needs to think that it’s of value to them, rather than you are being nagged to death.* (Patient, Female, 54).

For some patients, timing of the conversation was key. When preoccupied with other things such as illness they often did not want to think or talk about their weight. If the timing was right, however, the message was likely to be more impactful:

*Well, I found it was very useful that he mentioned it. It got me thinking [that] if I keep going, putting more weight, I won’t be able to move or walk. It’s true. I will need a walker if I keep going with my weight …*. (Patient, Female, 69).

Clinicians were more likely to initiate weight conversations, and approaches ranged from harsh and direct, introducing the topic sensitively, or being opportunistic. Clinicians frequently focused on risk assessment indicators (weight, calculating BMI, BP, or blood glucose level) to segue into discussions about lifestyle alterations. They tended to feel more comfortable if there was a physical imperative to intervene:

*I had a patient … in his early 40s where we do a health assessment and diabetes risk assessment............. So, I first did the risk assessment, and then said, ‘look you know, if you reduce your waistline from 112 to 107, and if you do regular exercise, your risk is reduced by 50%.’ Thankfully, he said straight away, ‘I know what needs to be changed, it’s just a bit of my eating habit.’ He has a sugar addiction; coke, soft drinks, and other things. So, he took it quite well* (FP, Male, Unknown).

Clinicians were also cognisant of the negative impact that stigma and judgement may have in their interactions with patients over weight management. Where present, these sentiments can drive a wedge between otherwise collaborating partners. It was regarded as important that clinicians remain supportive, accept failure in their patients, and are prepared to persevere when things do not go as planned. As one FP so eloquently stated:

*No-one likes being told what to do. You don’t tell them; you just help them to make their decision and you be there on their side in their journey. If they have failed, you don’t criticise them, but actually help them to stand up again and keep on going. The moment you start criticising; you know, you should have stayed on it, or you shouldn’t have done that, that’s the end of it. They will stop coming back to you, or they will come back, but they will no longer talk about those things. Such a judgmental attitude, … we all are told and taught [to avoid it], but still you see it a lot, even in the health field, … which is a bit sad.* (FP, Male, Unknown).

Low patient motivation as a barrier to weight management is widely reported. A provider’s perception of patient motivation, however, may not always align to that of the patient. Finding this shared ground can be the difference between a negative and a positive interaction, and any subsequent impact of this on communication may be the difference between pessimism and collaboration. Exploring opportunities to find this balance is essential:

*What I have personally experienced is, if the patient is very much motivated, then referring makes a really big impact. For example, doing the care plans and referrals for dietician and physios is when the person comes with that motivation and mindset; ‘yes, I want to make the change’. This is different to where the person is not that motivated, but opportunistically you have to bring it up. You discuss [weight] and try to explain the importance of weight loss and positive lifestyle changes and tell them that you are eligible for the care plan and Medicare funded program to get input from a dietician and physiotherapy. They will say, ‘yeah, yeah, I’ll do it, because I don’t have to pay.’ They’ll buy that thinking.* (FP, Male, Unknown).

Clinicians who were inclined to perceive patients as ‘unmotivated’ and ‘not prepared to change’ were less likely to engage with, or encourage, patients in weight management strategies. Overall, providers expressed an imperative to discuss weight with their patients. They understood patients will vary in their responses, that some will be more open than others, and, at times, the message will fall on deaf ears. At other times, however, the message might strike a chord and lead to something more positive:

*It’s a mixed bag, yes. A lot of them [patients] are very keen and a lot of them need persuasion. Some are reluctant, and some say I know I’m overweight, you don’t need to tell me that or whatever, but most are very engaged and realise that it is very important. So, I’m happy to discuss that [weight] in the beginning. I might list some of the benefits from weight reduction and exercise, some things that they might not be aware of; for example, cancer risk reductions, things like that. I’m trying to get them more on board to try and sell the strategy to them*. (FP, Male, 65+).

Patients also acknowledged that being motivated to change their thinking or behaviour was necessary, and was a major factor in their attitude towards weight loss:

*It’s got to come from within if I want to lose weight. It’s got to be something I want to do. I mean, people telling you to lose weight, it’s like my wife telling me lose weight, and I don’t always listen to her. I’ll be honest. I mean, people can talk to you every day nonstop, but if you don’t want to listen, no offence, you know you’re not going to change. You’ve got to want to change*. (Patient, Male, 62).

Motivation is not static and sometimes small gains can be seen as large gains by patients. In turn, this can lead to further motivation:

*My biggest issue is my weight and when I started doing this is when I finished work. In your mind’s eye you always lose lots and lots of weight but of course it doesn’t happen like that. I may not have lost a lot of weight, but I haven’t put it on either. So, for me, that’s a positive.* (Patient, Female, 62).

Some patients found motivation from participating in programs which emphasised consistency and monitoring. For one patient, anticipation of a call from the health coach provided considerable impetus towards meeting objectives and being ‘true to self’:

*I sort of fell off the wagon a little bit. She called me on Wednesday, I can’t lie to her, you know.*

*So, I’ve got to work harder in trying to lose weight and trying to do everything right before I speak to her so I’m not lying. It’s nothing really big, but it’s still a motivator.* (Patient, Male, 53).

The patient negativity that providers experience may be compounded because they do not have the skills to adapt their practices to elevate and/or engage the patient. In essence, the negativity is often reciprocal leading to minimal positive results around weight management. One FPN described working with a patient to identify solutions and how this could increase confidence and lead to enhanced patient motivation:

*So, for a lot of people there’s, ‘I can’t do it because … ’, so, they didn’t have the skills to work around that or have as many options perhaps as I might. So you say, ‘if you did this then and you could actually do it at night or with a friend or … have an exercise physiologist to tie it altogether. Or I’ve found that they just actually needed some physio first. Okay, so confidence is the issue, so let’s get that worked out and then we can do the rest … … ... some people just needed that little push to say, ‘yes, I’ve been talking about this and thinking about this for ages. Now that you’ve talked about it, we’ll do it.’* (FPN, Female, 45-54).

### Ensuring access to multi-modal weight management options that acknowledge differing levels of health literacy

Within this trial patients could take up two additional programs. The lifestyle app allowed them to set diet and lifestyle goals and then monitor their progress against these goals, whereas the telephone coaching option provided up to 10 coaching calls with a trained coach. There was, as expected, mixed feedback about these programs, with perhaps a slight preference for the coaching over the mobile app-based alternative.

Some simply did not engage with the app or found it too difficult to use. Others found it impersonal, or perceived it as not being interactive enough:

*Well, I basically thought it (mysnapp) was a waste of time. It’s just progress reporting and what you’ve actually done for the week. It’s a report card.* (Patient, Male, 72).

Conversely, some patients liked the app because it provided a constructive way to keep track of their diet and exercise, along with their progress around these goals:

*It [app] was just a good way of keeping track and make sure how many times a week you just done your bit of exercise. Make sure you’re keeping track of having your proper meals and how many serves of fruit or stuff like that you’re having.* (Patient, Male, 53).

Those who indicated a preference for the coaching service saw it as an opportunity to access regular contact and support:

*The phone calls meant more to me than the app because it was somebody actually encouraging you and listening to your story.* (Patient, Female, 63).

Whereas for others, the telephone coaching was too impersonal:

*I spent my working life on phones and emails, and I really just find them a bit – it overwhelms me. I hate email. … I’m retired and I only work part-time. I try and keep my life very, very simple. I like face-to-face.* (Patient, Female, 67).

Some providers preferred to refer patients rather than try to provide in-house management. This was an acknowledgement that they did not possess the skill set required to comprehensively manage the problem or, in the case of referral (particularly to a dietician or a sports physiologist), it was regarded as giving the patient access to the expertise they needed and would most benefit them.

Medication and bariatric surgery as potential and viable treatment options for weight management were raised by clinicians and patients alike. FPs were more inclined to report that their patients deferred to these options in lieu of trying weight loss programs or lifestyle changes. They were also cautious in case their patients became too focused on a ‘quick fix’ without thinking through the consequences:

*I’ve been having to advise my patients who are having bariatric surgery that this is only an adjunct. I say, ‘if you think that that will lose your weight automatically, it won’t.’ I think they’re all now hitting the gastric bypass which seems to be the most effective. But I might have seen two or three who, even with bariatric surgery, have kept their weight down, but over two or three years they haven’t. I’m happy to send people off [for surgery]. Let’s face it, when you’ve got a BMI of 45, you’re not going lose weight and so I think the surgery is reasonable. But I’m now really vetting them very hard.* (FP, Male, 65+).

It was evident from our data that, individually, patients developed firm preferences regarding approaches to weight management. They may have a general preference for exercise over diet, or develop preferences based on their social circumstances or previous experiences (both positive and negative). This preference might also relate to ‘personality’ and the degree to which the patient is ‘open’ to trying something new. Therefore, an intervention or program that is acceptable to, or which works for, one patient will not necessarily be acceptable to, or work for, another.

Our results also indicate that clinician awareness of each patient’s baseline understanding of their health is important, as is their understanding of each patient’s preferences for education, information, and instruction. In turn, some providers enjoyed assisting patients to improve their health literacy and considered it clearly within their remit:

*It’s amazing how people are really illiterate about their health. Often, we take it for granted because, the thing is, it’s in our head. Because it’s in our head, you think, you know, it’s in the other person’s head as well, but there is a big bridge to cross. That’s why I love general practice, you know, because we get these opportunities to know the patient dynamics and how they think and how to transfer that information across. Because patients often don’t say, ‘I don’t know doctor.’* (FP, Male, 35-44).

Many providers, however, found low levels of health literacy among patients to be challenging and too time intensive:

*I guess you have got to try to work in with general patients, try and talk to them at their level. Sometimes it’s hard to work out, you know, what someone’s level of literacy is.* (FP, Male, 55-64).

*I wouldn’t be able to go in-depth in terms of health literacy or consultation because we have limited time and we have to cover a whole lot of things, as in terms of health literacy, I don’t do a whole load of … information. … We didn’t spend that much time on it.* (FPN, Male, Unknown).

Patients with a more sophisticated understanding of their circumstances and health status did not appreciate being handed simple information on weight management, particularly if it was generic and did not resonate with them. They wanted new, innovative, and useable information that increased their knowledge of weight management strategies. In addition, many patients expressed a desire to improve their knowledge as they equated this with greater empowerment to take charge of their own care:

*I’m an avid reader anyway because he (FP) would always say things like, “Do you want the long version or the short version” to me because he knew I would read it. And so I look things up myself as well.* (Patient, Female, 71).

*It’s always good to gain new information and knowledge. … There might be a bit of curiosity to spend time … to get back into my fitness and that type of stuff. So, I think that was the main reasons to see if there were any new kits, tools, or strategies, or things like that that I should be focussing on that are out there.* (Patient, Male, 49).

## Discussion

This qualitative study indicates that delivering positive weight management in family practice with patients who are overweight or obese is challenged by the complexity of the task and the perceived motivation of patients. It is however facilitated by positive open communication and programs tailored to patient needs, preferences, and health literacy. The content of our themes firmly align with the findings of many other studies on obesity that identify the importance of positive patient-provider communication/relationships [23–25], recognise the impact of motivation in weight management and weight loss treatment [23, 26], acknowledge the importance of recognising individual health literacy levels and pitching education at that level [27, 28], and value tailoring programs to the patient’s particular needs [29].

Our study shows that clinicians can be very influential, in the context of a sustained, open, trusting, and therapeutic relationship. Primary care providers, whether FPs or FPNs, need enthusiasm, dedication, and to spend sufficient time with patients to understand their underlying concerns and tap into personal motivators effectively. This was reiterated throughout our study, where long-standing and person-centred relationships (interpersonal continuity) fostered an environment where weight could be better addressed in the context of preventive care [30, 31]. We know that patients often seek out and trust the advice they receive from their primary care providers [32, 33]. Patients in our study were more likely to be responsive to weight management advice if they perceived their provider as trustworthy, non-judgemental, empathetic, and respectful. Moreover, they reported being more inclined to engage in positive dialogue and attempt weight management and lifestyle behaviour change activities when interacting with a provider with whom they did not fear reprisal or judgment.

Concepts such as ‘patient noncompliance’ and low motivation often focus on patient failure, and the association between patient motivation and behaviour change is widely reported [34–36]. This was also evident within our study. FPs and FPNs often perceived their patients as lacking in insight, unmotivated, or unwilling. They often described them as presenting entrenched patterns of thinking and behaviour that were resistant to intervention. Conversely, some patients described being sensitive to feeling judged and stigmatised due to their weight. Understanding that patient readiness to change may alter over time, appreciating they may be influenced by past failures, and helping patients anticipate relapse can often improve patient satisfaction and lower clinician frustration during this process [37, 38]. Our study indicated that unless programs are perceived as relevant and valuable, patients are unlikely to try them or stay engaged with them. Moreover, personal circumstances (e.g. lack of time, financial stress, etc.) and other psychosocial factors will impact patient motivation and willingness and if not adequately addressed or accounted for, the patient is less likely to exhibit readiness to change. Adequately addressing psychosocial issues within this setting is challenging. Many people experience eating disorders, self-sabotaging behaviours, and poor body image. Equally many may suffer occupational and family stress, medical disorders and depression and/or anxiety, all of which can impede an individual from reaching and/or sustaining their weight loss goals. Currently FPs are encouraged to utilise multidisciplinary services such as exercise physiology, psychology, dietitians, and health coaches and consider therapeutic and surgical options after fully assessing patients [39].

Communication and clinical relationships that support continued collaboration between the patient and provider are valuable in supporting weight management [40]. However, they require personalised interventions to be successful [41]. As El Ghoch and Fakhoury [42] have commented, patients who are overweight or obese “know what to do, but also need to know how”. Many patients expressed that they found clear communication and a supportive clinical relationship with their health providers allowed them to ask more tailored questions on how they could manage their weight.

Identifying each patient’s level of understanding of their weight and health needs should inform the types of patient education materials, aids (e.g. apps) or health coaching being offered. Tailoring materials and programs to the patient’s readiness, circumstances, and health literacy may also help to increase patient motivation for behaviour change [20, 43]. Within this study, having informative and relevant instructions and education materials was perceived by providers as integral to guiding patients to follow through with advice, as was the availability of options and pathways for the patient and provider. Our results suggest that to achieve better results, patients need: a) individualised, achievable programs, b) programs that are assessed regularly and adapted according to the patient’s needs, and c) support from providers whom they respect and can impart the correct information effectively, manage their expectations and behaviours, and help them to stay motivated to change.

### Implications for family practice

While the views of patients and providers canvassed in this study further support the role of family practitioners in weight management and long-term continuity of care, they also highlight the significant challenges inherent in this endeavour. Family practice is an appropriate setting to address weight management over the long term, yet FPs/ FPNs may not be the only people to deliver weight loss interventions. Patients may access weight loss interventions through organisations such as Weight Watchers, formalised weight loss programs (e.g. Get Healthy), and via the Allied Health sector such as pharmacists and dieticians. Positioning family practice, and FPs specifically, as wholly responsible for guiding and managing patient weight may therefore be misplaced. It is reasonable to view family practice as the ‘starting point’ for weight management, but with recognition that additional supports in the form of referral, adjunctive medication, and surgery are viable options for some patients. Indeed, referral is a strategy used and preferred by many FPs [44, 45]. The family practice setting supports the development of longer-term therapeutic relationships with patients, which is further facilitated by good-quality communication between general practices and referral services. One alternative model is ‘shared care’ in which GPs and specialist services contemporaneously share in the care of patients supported by an understanding of each other’s roles, and effective mechanisms for communication and information sharing. Within our study, referring a patient on was a viable option for FPs who didn’t feel they had the depth of knowledge, skill or time to provide optimal treatment and advice to their patients. There is however a lack of clear referral pathways or management options for patients who are willing to engage in weight management [46], and inequity in access to coordinated surgical and specialist care [10]. As such, FPs and FPNs have a personal and continuing role in maintaining continuity of care even after referral. As a result, patients need to view the care provided by the referral services and the FP as consistent and reinforcing. A respectful and trusting provider-patient relationships underpins effective weight management. These relationships are facilitated by providers with the communication skills to initiate and guide ‘difficult conversations’ about weight and weight management with their patients, and the ability to build rapport with patients and to provide appropriate support. Although patients’ expectations vary, positive lifestyle messages skilfully delivered at the right time and with the right sentiments can have a notable impact on patient motivation. In turn, this can lead to enhanced patient engagement in behaviour change and/or acceptance of a referral to another provider or service (e.g. a dietician, health coach, or exercise physiologist).

A primary challenge for FPs and FPNs in implementing weight management programs, including patient education on the health risks of their lifestyle behaviours, is the need to manage time constraints and balance competing demands [33]. Put simply, if family practices are not funded adequately, they do not have the time to provide effective education and support. Family practices that support and nurture patients provide them with the capability and opportunity to achieve a healthy weight [47]. FPs and FPNs need to stay involved in their patient’s weight management journey, even though this is often difficult due to time, capacity, and/or funding constraints. Even if patients do not accept help at first, family practices need to prioritise weight management, demonstrate empathy towards the patient’s situation, attempt to impart the correct information, and keep checking in with their patients to assess whether their acceptance for, or readiness to change has shifted. These practices should all be performed while reiterating the need for the patient to continue to attend the family practice.

### Study strengths and limitations

The findings reported here should be considered within the strengths and limitations of the study. Notably, these findings emerged in the context of a quantitative randomised controlled trial where participants in the intervention group ‘selected themselves’ for participation in the qualitative phase. Although our patient sample was broadly representative of the intervention group, all were interviewed in English and two thirds were born in Australia, so they are not necessarily representative of the general population. All interviews were conducted by telephone and were reasonably short (average 23 minutes), however these were highly focused and were offered via telephone due to the geographic dispersion of the participants. Performing the patient interviews face-to-face or via a video and audio-based communication software may have provided more non-verbal communication data which was not available through phone interviews. It is possible that patients who agreed to be in the qualitative study were somewhat more engaged or had undergone a better experience with the intervention. Our sample also was drawn from four urban/urban fringe practices in Adelaide and Sydney and therefore may not be generalisable to all general practices. These factors must be considered when interpreting the implications of the findings for practice and, ultimately, the generalisability of any practice recommendations. We received various responses from patients, both positive and negative, which indicates that our sample included patients across the weight loss spectrum.

It is generally accepted that qualitative research permits the utilisation of relatively smaller samples [48]. Our sample of FPs and FPNs was drawn from two states but only from four family practices. Because we approached practices that had been more successful in recruiting patients, they potentially represent higher performing intervention practices generally. We should not assume that the views of these providers are therefore indicative of the views of all FPs and FPNs. We do note, however, that the themes identified from the provider data align well with the findings of other qualitative studies which included interviews with providers for their views and perceptions related to weight management in family practice.

## Conclusion

Providing weight management within the environment of family practice is complex. Targeting obesity with individuals requires commitment and a good therapeutic relationship, and an acceptance that in some cases only small gains will be achieved. Ongoing support of family practice is required through funding and policy changes if they are to provide comprehensive weight management to patients that is both timely and effective. The programs that are more likely to work; namely, those that seek to engage patients and target motivations should be highly individualised and tailored, relevant to each patient, regularly monitored by health providers, and delivered within an environment that fosters mutual respect and trust. When targeting behaviour change, family practitioners must therefore ensure that all communications with patients are tailored to the level of health literacy of the patient, as well as consistent and positive in their messaging. This will help to engage the patient and foster supportive relationships while acknowledging obesity as a chronic relapsing condition with dynamic influences.

## Supplementary Information

**Additional file 1.**

## Data Availability

The data that supports the findings of this study are available on request from the corresponding author, KP. This qualitative data is not publicly available because it contains information that could compromise the privacy of research participants.

## Abbreviations

BP: Blood pressure
BMI: Body mass index
FPN: Family practice nurse
FP: Family practitioner
WC: Waist circumference

## Footnotes

1 Further information about the service can be accessed at https://www.gethealthynsw.com.au/
