# Supplementary material for: In it for the long haul: the complexities of managing overweight in family practice: qualitative thematic analysis from the Health eLiteracy for Prevention in General Practice (HeLP-GP) trial
Source: BMC Prim Care. 2023 Feb 27;24:57. doi: 10.1186/s12875-023-01995-w (PMC9972770; doi:10.1186/s12875-023-01995-w)
Supplement: Supplementary file 1 — Additional file 1. [file 12875_2023_1995_MOESM1_ESM.docx]

# Appendices

## Appendix A: Patient Qualitative Interview Guide

*My name is (13) and I am a research assistant from the University of New South Wales and working on the HeLP-FP study that you have been participating in at [insert practice name]. Would now be a good time to have a conservation about the HeLP-FP study?*

If patient is busy: *Would there be a better time to call you back?*

If patient is happy to proceed*:* *Our conservation today is private and confidential. Would it be okay if I record today’s conversation?*

If patient says no, explain that the recording is just for our university research team and not your practice. If patient still says no: *Would you be okay if we took notes instead?*

If the patient asks about time: *This interview should last no more than 20-30 minutes.*

Preamble:

*The research team are interested in understanding what patients think about preventive care. What do you understand is meant by this term?*

Prompts: What does it mean to try to prevent something from happening?

*When we talk about preventive care, we mean regular healthcare that includes things like screening, check-ups and conversations with your doctor or nurse about ways to prevent you from developing a health problem.*

*I am going to ask you some questions but what I would like is for us to have a conversation. Do you have any questions before we begin?*

[Notify patient that you will now start the recording and confirm verbally that they are happy to participate in the interview]

**Questions (*prompts in italics)***

Some people often have different views on how much of this type of care they want from their Family practitioner or practice nurse. For example, some might want their doctor to regularly weigh them and provide advice on their diet and exercise, while others may not want this type of advice at all.

1. **What are your thoughts on this type of care?**

*Prompts: Would you like this advice? / Is this something that is important to you? / Would you like more or less of this type of care?*

We now want to know about your experience of preventive care:

1. **Have you seen a FP or practice nurse in the past 6 months? Can you tell me about your visit?**

*Prompts: Did you receive any health checks? / Did they discuss with you diet, exercise or weight? / What sort of health assessments were done in this session (waist circumference, blood pressure etc.)?*

1. **Did you find these conversations useful? How did you feel about the health check process?**
2. **Have these conversations led to changes in your lifestyle? Can you tell me a bit more about this?**
3. **Is there anything that has:**
4. **helped you discuss preventive care with your general practitioner/practice nurse?**
5. **stopped you discuss preventive care with your general practitioner/practice nurse?**

*Prompts: Time spent talking about other things/interest / Willingness of the patient or health practitioner to discuss these issues*.

1. **Are there any ways you think your FP/ PN could improve how they deliver preventive care?**

*Prompts: What does your FP/PN do well/ What could the FP/PN do better?*

1. **Were you referred to the telephone coaching service Get Healthy? Can you tell me a bit about this experience?**

*Prompts: Are you still participating?*

1. **We are also interested in whether using mobile phone apps might be useful in preventive care. As part of the HeLP project, we developed the *mysnapp* app. Were you offered the *mysnapp* app? Can you tell me a bit about your experience?**

*Prompts:*

Patients who did not take up the app: *What were the reasons you did not/ could not participate?*

For patients who used the *mysnapp*app: *In what ways did you use mysnapp?*

1. **What was the least and most useful thing about *mysnapp*? Are you still using it? How long did you use it?**
2. **Do you think using the app has influenced how you interact with your Family practitioner and practice nurse? If yes, how? If not, why not?**

*Prompts: Did you show FP/PN the app during consultation? / Did you discuss the information*

*collected/monitoring/progress?*

*Now we want to ask you about the HeLP-FP project overall* (*mysnapp* app, the conversations with your FP and practice nurse and the telephone coaching from Get Healthy).

1. **Do you think your view of preventive care has changed over the past 6 months while you were involved in the study?**

*Prompts: What has impacted this or influenced the way you think about preventive care*

(*i.e., family/other people/an experience, changes to your lifestyle etc.)?*

1. **Why did you decide to participate?**

*Prompts: Motivation / Change behaviour / Lifestyle*

1. **What did you find most useful/ least useful about the study?**
2. **Would you recommend the HeLP-FP study to a friend?**

## Appendix B: Family Practitioner Qualitative Interview Guide

To be conducted with selected general practitioners and practice nurses in the intervention group following the intervention.

- Introduce yourself
- Estimated duration of interview
- Provide overview of the interview (semi-structured interview intended to give general direction and an overview of the potential question routes)
- Provide process for the discussion (audio recording, taking notes, etc…)
- Consent for recording
- Ask if they have any questions or need any clarification

Preamble:

*This interview will collect feedback on your experience of participating in the HeLP-FP study plus your thoughts regarding the level of involvement of your patients. HeLP-FP sought to explore whether a preventive intervention in which participating patients were encouraged to address lifestyle changes (diet, physical activity) resulted in an improvement in overall health or changes in behaviour.*

*We are also interested in understanding the implementation of preventive care for overweight and obese patients in your practice and in Family practice more widely.*

1. **a) What is your experience of delivering preventive care/lifestyle advice to overweight and obese patients generally? For example: Can you describe a recent consultation when you discussed diet and physical activity with a person who was overweight or obese?**

*Prompts: What did you do/what was discussed? / Who initiated the discussion and why? / Did you feel equipped or prepared to have the discussion? / What was the outcome of the discussion?*

If the respondent did not outline a difficult experience:

**b) Can you outline a consultation where discussing preventive care proved difficult?**

*Prompts*: *Why was it difficult? / How did the patient react? / What was the outcome of the discussion?*

1. **a) Have you come across the term ‘health literacy’ before?**

If respondent answers ‘yes’, go to Question 2b

**b) What do you understand by this term?**

If respondent answers ‘no’, read the following definition and then go to Question 2c:

Health literacy can be described as the *capacity to acquire, understand, and use information in ways which promote and maintain good health*.

**c) How do you think health literacy impacts your consultations around prevention?**

**d) How does low health literacy influence your approach to patients?**

*Prompts*: *Communication (e.g., asking patients to repeat what they have heard (teach-back) and refining the conversation based on this; using an interpreter) / Helping patients to navigate to referral services / Providing additional educational material / Follow up recall sent through the practice software*

**I now want to ask you some questions about your experience of the HeLP-FP project in particular.**

1. **a) You may have had one or more patient consultations as part of the HeLP-FP project. Can you tell me about one of these consultations?**

*Prompts*: *Did you have any difficulties getting patients to attend? / What did you do during the health check (e.g., BP. weight, WC, BMI)? / What did you discuss during the consultation? Did you provide advice, were you able to set goals with the patients? / How did the patient respond to the health check? / Did you find the consultation effective? / Can you think of any ways in which these types of consultations could be improved?*

**b) We designed the health check around the idea of the 5A’s model of behaviour change. This refers to Ask/Assess, Advise/Agree, Assist, and Arrange. Are you familiar with this model?**

*Prompts*: *Do you incorporate any of these aspects into your consultations with overweight and obese patients (e.g., do you consider factors influencing patients’ lifestyle as part of assessing risk)? / Are there any challenges to using the 5As with your patients (e.g., motivation of patients, level of understanding of patients, time, need for consistency/review)?*

**c)** **Did you arrange any follow-up visits as part of the HeLP-FP study?**

If yes:

*Prompts: What worked well? / What did not work well? / What factors influenced this (e.g. practice, nurse, patient, other factors)?*

If no:

*Prompts: What barriers did you experience (e.g. time, lack of interest by patient, non-attendance by patient)? / Employed just to do health checks*

1. **a) Within the HeLP-FP study patients could elect to have an app (*mysnapp*) and/or be referred to Get Healthy telephone coaching. Did you discuss *mysnapp* with patients?**

*Prompts: Did they install it? / Did they have any issues using it or instructing patients on how to use it? / Did they enter any data? / Did they use it as a tool in subsequent consultations? / What was the patient’s response to the app (e.g., enthusiastic, reluctant, already using apps, poor literacy with apps)? / Any general comments about the app?*

**b) Did you refer patients to the Get Healthy service?**

*Prompts: Did you have any issues with the referral process? / What was the patient’s response to the referral? / What was the patient’s feedback on the service? / Any general comments about the Get Healthy service?*

**c) Has being in this study influenced the way you approach preventive care with overweight or obese patients?**

**5. Is there anything else you would like to share or discuss regarding preventive care or the HeLP-FP study?**

*Prompts: Recommendations for future projects / Experience of being a casual nurse in the project*

**Thank you very much for your time, both during this interview and the HeLP-FP study overall.**

## Appendix C: Family Practice Nurse Qualitative Interview Guide

To be conducted with selected general practitioners and practice nurses in the intervention group following the intervention.

- Introduce yourself
- Estimated duration of interview
- Provide overview of the interview (semi-structured interview intended to give general direction and an overview of the potential question routes)
- Provide process for the discussion (audio recording, taking notes, etc…)
- Consent for recording
- Ask if they have any questions or need any clarification

Preamble:

*This interview will collect your feedback and experience of participation in the HeLP-FP study plus your thoughts regarding the involvement of your patients. HeLP-FP sought to explore whether a preventive intervention in which participating patients were encouraged to address lifestyle changes (diet, physical activity) resulted in an improvement in overall health or changes in behaviour.*

*We are also interested in understanding the implementation of preventive care for overweight and obese patients in your practice and in Family practice more widely.*

1. **a) What is your experience of delivering preventive care/lifestyle advice to overweight and obese patients generally? For example: Can you describe a recent consultation when you discussed diet and physical activity with a person who was overweight or obese?**

*Prompts: What did you do/what was discussed?/ Who initiated the discussion and why? / Did you feel equipped or prepared to have the discussion?/ What was the outcome of the discussion?*

If the respondent didn’t outline a difficult experience:

**b) Can you outline a consultation where discussing preventive care proved difficult?**

*Prompts: Why was it difficult? / How did the patient react? / What was the outcome of the discussion?*

1. **a) What enables you to provide preventive care/lifestyle advice to your patients who are overweight or obese?**

*Prompts: Motivated patients / Extensive experience / Knowledge / Adequate time / Confidence / Positive attitude towards preventive care / Strong focus on prevention by Family practice / Employed for the study so primary role was the health check*

**b) What barriers have you experienced in providing preventive care/lifestyle advice to your patients who are overweight or obese?**

*Prompts: Lack of experience / Lack of broad knowledge / Too busy / No appropriate consultation facilities / lack of interest or motivation from the patient / Lack of support from management / Not employed by the practice*

**c) How important do you view your role in providing advice on preventive care/lifestyle advice to patients who are overweight or obese at your practice/within Family practice?**

*Prompts: Do you think it is another health professional’s role? / Do you view preventive care as a low priority compared to acute treatment? / Is it vital as you have the best rapport with the patient to have these conversations?*

**d) Do you feel preventive care is valued in your practice/ at the practice where you conducted the health checks? If yes, why? If no, why?**

1. **a) Have you come across the term ‘health literacy’ before?**

If respondent answers ‘yes’, go to Question 2b

**b) What do you understand by this term?**

If respondent answers ‘no’, read the following definition and then go to Question 3c:

Health literacy can be described as the *capacity to acquire, understand, and use information in ways which promote and maintain good health*.

**c) How do you think health literacy impacts your consultations around prevention?**

**d) How does low health literacy influence your approach to patients?**

*Prompts: Communication (e.g. asking patients to repeat what they have heard (teach-back) and refining the conversation based on this; using an interpreter) / Helping patients to navigate to referral services / Providing additional educational material / Follow up recall sent through the practice software*

**I now want to ask you some questions about your experience of the HeLP-FP project in particular.**

1. **a) You may have had one or more patient consultations as part of the HeLP-FP project. Can you tell me about one of these consultations?**

*Prompts: Did you have any difficulties getting patients to attend? / What did you do during the health check (e.g., BP, weight, WC, BMI)? / What did you discuss during the consultation? / Did you provide advice, were you able to set goals with the patients? / How did the patient respond to the health check? / Did you find the consultation effective? / Can you think of any ways in which these types of consultations could be improved?*

**b) We designed the health check around the idea of the 5As model of behaviour change. This refers to Ask/Assess, Advise/Agree, Assist, and Arrange. Are you familiar with this model?**

*Prompts: Do you incorporate any of these aspects into your consultations with overweight and obese patients (e.g. do you consider factors influencing patients’ lifestyle as part of assessing risk)? / Are there any challenges to using the 5A’s with your patients (e.g. motivation of patients, level of understanding of patients, time, need for consistency/review)?*

**c)** **Did you arrange any follow-up visits as part of the HeLP-FP study?**

If yes:

*Prompts: What worked well? / What did not work well? / What factors influenced this (e.g. practice, nurse, patient, other factors)?*

If no:

*Prompts: What barriers did you experience (e.g. time, lack of interest by patient, non-attendance by patient)? / Employed just to do health checks*

1. **a) Within the HeLP-FP study patients could elect to have an app (*mysnapp*) and/or be referred to *Get Healthy* telephone coaching. Did you discuss *mysnapp* with patients?**

*Prompts: Did you install it? / Did you have any issues using it or instructing patients on how to use it? / Did you enter any data? / Did you use it as a tool in subsequent consultations? / What was the patient’s response to the app (e.g. enthusiastic, reluctant, already using apps, poor literacy with apps)? / Any general comments about the app?*

**b) Did you refer patients to the *Get Healthy* service?**

*Prompts: Did you have any issues with the referral process? / What was the patient’s response to the referral? / What was the patient’s feedback on the service? / Any general comments about the Get Healthy service?*

**c) Has participating in this study influenced the way you approach preventive care with overweight or obese patients?**

**6. Is there anything else you would like to share or discuss regarding preventive care or the HeLP-FP study?**

*Prompts: Recommendations for future projects / Experience of being a casual nurse in the project*

**Thank you very much for your time, both during this interview and the HeLP-FP study overall.**

## Appendix D: COREQ 
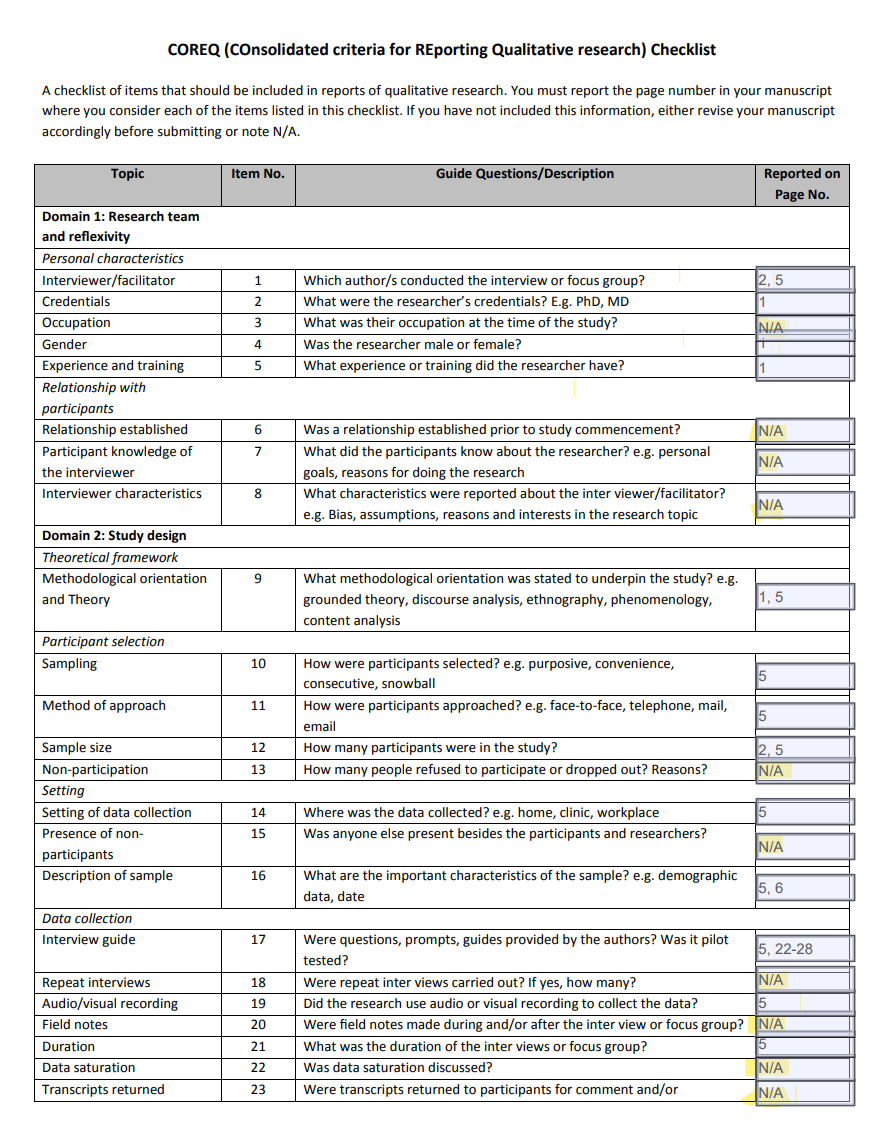


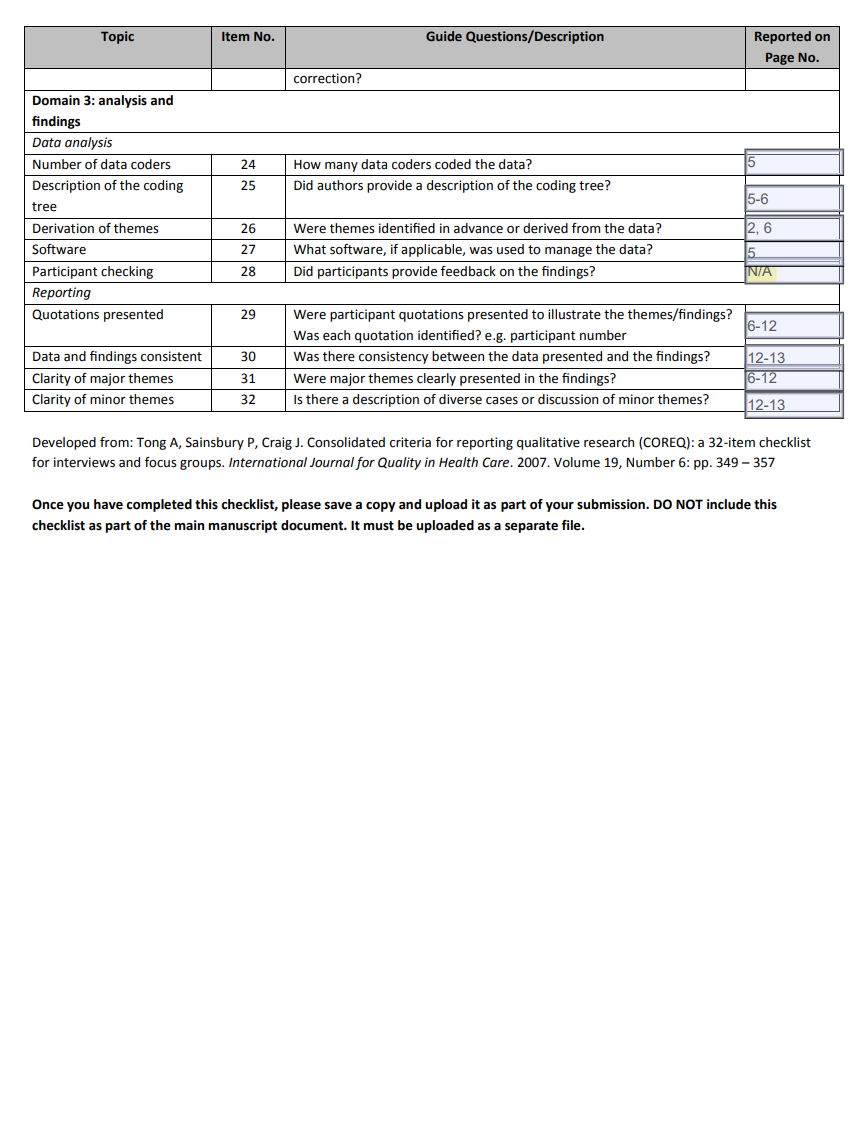


**1.5 Appendix E: Stages of qualitative analysis (Braun & Clarke, 2006)**

| **Stage** | **Description of approach** | **Researchers** |
| --- | --- | --- |
| Familiarisation | Transcripts were read and re-read, and the researchers became intimate and familiar with the dataset content. Familiarisation with, and orientation of, data took place by listening to the interview and recording memos and notes on any analytic ideas and insights related to each data item firstly and then as a whole dataset. | KP, SP |
| Coding | Key themes were identified and explored systematically throughout the dataset. Code labels (analytically meaningful descriptions) were developed according to our analytical take of the data item. | KP, SS, SP, EDW |
| Generating themes | Emergent themes and shared patterns of meaning were then identified across the dataset. Codes were clustered (shared idea or concept). Candidate-based themes were then developed and subsequently discussed more broadly with researchers specialised in qualitative research. | KP, SP, SS, EDW, MH |
| Developing and reviewing themes | By comparing and contrasting potential themes (central organising concepts), recurring themes and discrete categories were identified across the data set. | KP, SP, SS, EDW, MH |
| Refining, defining and naming themes | Themes were fine-tuned and named to develop an agreed set of themes (critical framing). The naming of themes was developed and refined over several months. The process went from six emergent themes to five themes with an overarching theme, which were then consolidated and collapsed to three themes. | KP, SP |
| Write up | Informal writing of the process occurred throughout the procedure. Formal write up occurred at the end, weaving together our analytic narrative, vivid data extracts, and a story to form this paper. | KP, SP |
